# Resistance evolution of hypervirulent carbapenem-resistant *Klebsiella pneumoniae* ST11 during treatment with tigecycline and polymyxin

**DOI:** 10.1080/22221751.2021.1937327

**Published:** 2021-06-13

**Authors:** Xi Jin, Qiong Chen, Fang Shen, Yan Jiang, Xueqing Wu, Xiaoting Hua, Ying Fu, Yunsong Yu

**Affiliations:** a Department of Infectious Diseases, Sir Run Run Shaw Hospital, Zhejiang University School of Medicine, Hangzhou, People’s Republic of China; b Key Laboratory of Microbial Technology and Bioinformatics of Zhejiang Province, Hangzhou, People’s Republic of China; c Regional Medical Center for National Institute of Respiratory Diseases, Sir Run Run Shaw Hospital, Zhejiang University School of Medicine, Hangzhou, People’s Republic of China; d Department of Clinical Laboratory, Affiliated Hangzhou First People’s Hospital, Zhejiang University School of Medicine, Hangzhou, People’s Republic of China; e Department of Clinical Laboratory, The second Hospital of Shaoxing, Shaoxing, People’s Republic of China; f Department of Clinical Laboratory, Sir Run Run Shaw Hospital, Zhejiang University School of Medicine, Hangzhou, People’s Republic of China

**Keywords:** Carbapenem-resistant *Klebsiella pneumoniae*, hypervirulence, tigecycline resistance, colistin resistance, in-host, whole-genome sequencing

## Abstract

Hypervirulent carbapenem-resistant *Klebsiella pneumoniae* (hv-CRKP) has recently aroused increasing attention, especially ST11, the predominant CRKP clone in China. Here, we report a case of hv-CRKP-associated infection and reveal the in-host evolution of its mechanism of resistance to tigecycline and polymyxin under clinical therapy. A total of 11 *K. pneumoniae* carbapenemase (KPC)-producing CRKP strains were consecutively isolated from a male patient who suffered from continuous and multisite infections. String and antimicrobial susceptibility tests identified seven hypermucoviscous strains and three tigecycline-resistant and four colistin-resistant strains. *Galleria mellonella* larvae infection model confirmed the hypervirulence. Pulsed-field gel electrophoresis (PFGE) separated three PFGE clusters among all strains, and further Southern blotting detected that *bla*_KPC-2_ was located on the same-sized plasmid. Whole-genome sequencing showed that all strains belonged to the hv-CRKP ST11-KL64 clone. Diverse hypervirulence factors and resistance genes were identified. Further sequencing with the Nanopore platform was performed on the CRKP-Urine1 strain, which contained one virulence plasmid (pVi-CRKP-Urine1) and two resistance plasmids (pKPC-CRKP-Urine1 and pqnrS1-CRKP-Urine1). The gene mutations responsible for tigecycline or colistin resistance were then amplified with PCR followed by sequencing, which indicated that mutations of *ramR* and *lon* were the potential loci for tigecycline resistance and that the *pmrB*, *phoQ* and *mgrB* genes for colistin resistance. A novel frameshift mutation of *lon* was identified in the high-level tigecycline-resistant strain (MIC, 128 mg/L). The results indicate that the hypervirulent ST11-KL64 clone is a potential threat to antiinfection treatment and is capable of rapid and diverse evolution of resistance during tigecycline and polymyxin treatment.

## Introduction

Infectious disease caused by carbapenem-resistant *Klebsiella pneumoniae* (CRKP) has been an urgent concern to health-care institutes because it is associated with high mortality and morbidity [1]. Epidemiological studies have revealed that clonal complex 258 (CC258) is one of the primary CRKP clones disseminating throughout the world [2,3], among which sequence type 11 (ST11) is the predominant hospital-acquired clone in China [4]. Notably, an increasing number of studies have reported that ST11 CRKP is capable of developing resistance to antimicrobial agents and carries virulence plasmids, which benefit its survival in the host [5,6].

In terms of the multidrug resistance of CRKP isolates, tigecycline and polymyxin play a vital role in clinical practice as the “last-line” antibiotics for CRKP infections [7–10]. However, additional counterpart resistance issues emerge during clinical treatment and cause great challenges to public health [11–13]. The resistance mechanisms of CRKP to tigecycline have been uncovered predominantly by the overexpression of resistance-nodulation-cell division (RND) efflux pumps (e.g. AcrAB and OqxAB) or the deactivation of efflux pump negative regulators [14]. Other determinants, such as plasmid-borne *tet*(A) variant genes and mutated *rpsJ* genes, contribute to tigecycline insusceptibility (intermediate and resistance) as well [15,16]. Acquired colistin resistance is related to genetic alterations in lipid A modification, including the overexpression of two-component regulatory systems (PmrAB and PhoPQ), inactivation of the MgrB protein and carriage of *mcr-1*-harboring plasmids [17,18].

Here, we reported that a 68-year-old patient who had complaints of fever and chills for over 24 h was first admitted to the Affiliated Hangzhou First People's Hospital, Zhejiang University School of Medicine, and then transferred to Sir Run Run Shaw Hospital. During the two sequential hospitalizations, he experienced metastatic and severe infections caused by CRKP even under long-term antibiotic treatments, including carbapenems, tigecycline, polymyxin B and ceftazidime/avibactam. On the basis of whole-genome sequencing and bioinformatic analysis, we revealed the evolution strategy of CRKP in developing resistance to tigecycline and colistin.

## Materials and methods

### Medical history of infection

On October 8th, 2018, the patient was admitted to the hospital and diagnosed with urinary tract infection and bloodstream infection (BSI) on the fourth day of admission when two CRKP isolates (named CRKP-Urine1 and CRKP-Blood1) were isolated from urine and blood samples, respectively. A tigecycline and carbapenem (meropenem or imipenem) combination was used immediately to treat the infections, but he still developed a renal abscess on the 11th day. On the 40th day, the BSI recurred, and two CRKP isolates (CRKP-Blood2 and CRKP-Blood3) were collected consecutively. The antimicrobial therapy was immediately changed to polymyxin B in combination with tigecycline, but another two CRKP isolates (designated CRKP-Urine2 and CRKP-Pus1) were cultured from a urine sample and cutaneous abscess of the lower limb on the 50th day, which indicated that the infection had not yet been controlled.

The patient was then transferred to Sir Run Run Shaw Hospital on November 28th for further treatment. The antimicrobial agent ceftazidime/avibactam was used on the second admission day when two CRKP isolates (CRKP-Pus2 and CRKP-Pus3) were identified from the abscess samples of the right lower limb on the 2nd and 7th days, respectively. The patient received intestinal CRKP screening on the first and second days of admission, both of which were positive for CRKP (CRKP-Feces1 and CRKP-Feces2). The CRKP infections were completely controlled after 20 days of ceftazidime/avibactam usage. The patient was finally discharged on December 18th, whereas one CRKP strain (CRKP-Feces3) was isolated from fecal samples on January 14th, 2019.

**Figure 1.**
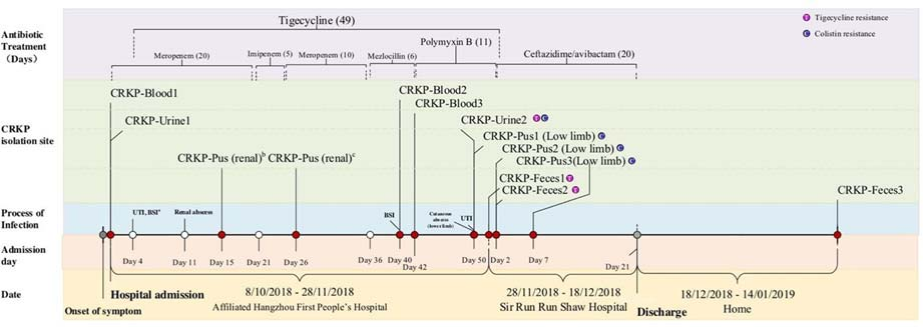
Medical history of the patient. Note: ^a^ UTI, urinary tract infection; BSI, bloodstream infection. ^b, c^ The CRKP isolates were not stored.

The medical history and antibiotic stewardship of the patient and the counterpart isolates are summarized in Figure 1.

### Species identification, antimicrobial susceptibility testing, virulence assay and detection of carbapenem-resistant genotype

Bacteria were isolated from clinical samples according to standard culture and isolation procedures as previously reported [19]. A Vitek 2 system (bioMérieux, France) was used for species identification and antimicrobial susceptibility testing (AST) in accordance with the manufacturer's instructions. The agar dilution method was used to determine the minimum inhibitory concentrations (MICs) of imipenem, meropenem, ceftazidime/avibactam and fosfomycin, and the broth microdilution method was used for colistin and tigecycline according to the standard protocols of the Clinical and Laboratory Standards Institute (CLSI) guidelines [20]. The results of AST were interpreted on the basis of the breakpoints of the CLSI except for tigecycline, which followed the criteria of the USA Food and Drug Administration (FDA) (https://www.fda.gov/drugs/development-resources/tigecycline-injection-products).

The string test was used to detect the hypermucoviscous phenotype [5]. To further detected the virulence potential, *Galleria mellonella* larvae infection model was performed on four-pair of strains from all infection sites following modified previous protocol [5]. Healthy larvae were purchased from Tianjin Huiyude Biotech Company (Tianjin) and weighing at a range of 250∼300 mg. An hvKP reference strain NTUH-K2044 (NC_012731.1) and a classic *K. pneumionae* strain KP04017 (RXNG00000000) identified in previous study were used as the positive and negative controls, respectively [21,22]. Each group contained ten larvae and were tested in triplicate experiments. After a predetermined appropriate inoculum of 1×10^6^ CFU of bacteria, survival of larvae was measured every 4 h for one day.

PCR and sequencing were used for the confirmation of carbapenem resistance genes [23].

### Pulsed-field gel electrophoresis and southern blotting analysis

PFGE was performed to assess the clonal relationship of the CRKP isolates, and the same clone was recognized within three-fragment differences in PFGE patterns [24]. S1-digested PFGE and Southern blotting were utilized to confirm the location of the *bla*_KPC-2_ gene [25].

### Whole-genome sequencing and sequence analysis scheme

Genomic DNA of all strains was extracted with a QIAamp DNA mini kit (Qiagen Valencia, CA) and sequenced by using the Illumina HiSeq X Ten platform (Illumina, San Diego, CA), with a paired-end mode of 2×150 bp, and contigs were assembled as previously described [26]. MinION sequencing (Oxford Nanopore Technologies Inc., UK) was further performed on the isolates CRKP-Blood1, CRKP-Urine1, CRKP-Urine2, and CRKP-Feces3. Complete genomes were generated by the Unicycler v0.4.0 tool [27] and annotated with the prokka 1.11 tool [28].

Multilocus sequence typing (MLST) and acquired antimicrobial resistance genes were identified by using the online CGE tool (https://cge.cbs.dtu.dk). Phylogenetic relationships were evaluated by core genome multilocus sequence typing (cgMLST) analysis, followed by generation of a minimum-spanning tree by using SeqSphere+ software (Ridom GmbH, Muenster, Germany) [29]. Virulence genes were predicted by using the online tools VFanalyzer (http://www.mgc.ac.cn/cgi-bin/VFs/v5/main.cgi?func=VFanalyzer) and Kaptive Web (http://kaptive.holtlab.net/). BLASTn was used for homologous comparisons of chromosomes and plasmids to the sequences in the NCBI database (https://blast.ncbi.nlm.nih.gov/Blast.cgi). The results of sequence alignment comparison, features of resistance and virulence factors, and base composition plots were depicted in graphic maps by the CGView server (http://stothard.afns.ualberta.ca/cgview_server/) [30].

The sequences of CRKP strains were submitted to database of NCBI (National Center for Biotechnology Information) with the accession numbers of PRJNA664283 (in progress).

### Confirmation of mutated genes associated with tigecycline or colistin resistance

Tigecycline resistance genes included efflux pump-related genes (*acrA/B/R*, *oqxA/B/R*, *rarA*, *ramA/R*, *marA/R*, *soxS/R* and *lon*), *tet* genes [*tet*(A), *tet*(X), *tet*(L) and *tet*(M)], and the ribosomal protein S10-encoding gene (*rpsJ*) [15,31,32], and colistin resistance genes were focused on LPS modification genes (*pmrA/B*, *phoP/Q*, *crrA/B*, *mgrB* and *mcr-1*) [17]. By using CRKP-Urine1 as the reference sequence, tigecycline or colistin resistance genes were involved in the sequence analysis on the basis of the long contigs obtained by Illumina sequencing. All identified mutations were then confirmed by PCR and sequencing with the primers shown in Table S1.

### Ethics statement

The study was approved by the local Research Ethics Committee of Sir Run Run Shaw Hospital with a waiver of informed consent (Approval No.20191231-20).

## Results

### Phenotypes of antimicrobial resistance and virulence of CRKP isolates

Eleven CRKP strains showed similar AST profiles, which were resistant to most of the tested antibiotics but were susceptible to ceftazidime/avibactam (Table 1). Three tigecycline-resistant (CRKP-Urine2, CRKP-Feces1 and CRKP-Feces2) and four colistin-resistant strains (CRKP-Urine2, CRKP-Pus1, CRKP-Pus2 and CRKP-Pus3) were identified (Table 1 and Figure 1). Seven strains showed the hypermucoviscous phenotype (Table 1). Further *G. mellonella* larvae infection model assay showed that all test strains were relatively more virulent than classic strain KP04017. Notably, six strains showed higher virulence level than hvkp reference strain NTUH-K2044. No significant survival differences were found between the resistant strains and their parent strains. (Figure S1).

**Table 1.** Phenotype detection results from virulence and antimicrobial susceptibility testing.

Name	String test^a^	Antimicrobial susceptibility result (MICs, mg/L)^b^															
		Vitek 2 system										Agar dilution				Broth dilution	
		TZP	CTT	CAZ	FEP	AMK	GEN	TOM	CIP	LEV	NIT	IMP	MEM	FOS	CAZ/AVI	TGC	CST
CRKP-Urine1	**+**	**≥128**	**≥64**	**≥64**	**≥64**	**≥64**	**≥16**	**≥16**	**≥4**	**≥8**	**≥512**	**128**	**>128**	**>1024**	4/4	4	<0.03
CRKP-Blood1	**+**	**≥128**	**≥64**	**≥64**	**≥64**	**≥64**	**≥16**	**≥16**	**≥4**	**≥8**	**≥512**	**128**	**>128**	**>1024**	2/4	4	<0.03
CRKP-Blood2	**+**	**≥128**	**≥64**	**≥64**	**≥64**	**≥64**	**≥16**	**≥16**	**≥4**	**≥8**	**≥512**	**128**	**>128**	**>1024**	2/4	4	<0.03
CRKP-Blood3	**+**	**≥128**	**≥64**	**≥64**	**≥64**	**≥64**	**≥16**	**≥16**	**≥4**	**≥8**	**256**	**128**	**>128**	**1024**	4/4	4	<0.03
CRKP-Urine2	–	**≥128**	**≥64**	**≥64**	**≥64**	≤2	≤1	≤1	**≥4**	**≥8**	**≥512**	**128**	**>128**	32	2/4	**8**	**> **
CRKP-Pus1	**+**	**≥128**	**≥64**	**≥64**	**≥64**	**≥64**	**≥16**	**≥16**	**≥4**	**≥8**	**≥512**	**128**	**>128**	**>1024**	1/4	4	**128**
CRKP-Feces1	–	**≥128**	32	**16**	**32**	**≥64**	**≥16**	**≥16**	**≥4**	**≥8**	**≥512**	**64**	**128**	**512**	<0.06/4	**16**	2
CRKP-Pus2	**+**	**≥128**	**≥64**	**32**	**≥64**	**≥64**	**≥16**	**≥16**	**≥4**	**≥8**	**≥512**	**128**	**>128**	**>1024**	2/4	4	**128**
CRKP-Pus3	**+**	**≥128**	**≥64**	**≥64**	**≥64**	**≥64**	**≥16**	**≥16**	**≥4**	**≥8**	**256**	**128**	**>128**	**1024**	4/4	2	**64**
CRKP-Feces2	–	64	**≥64**	**16**	**32**	**≥64**	**≥16**	**≥16**	**≥4**	**≥8**	**256**	**64**	**>128**	**>1024**	2/4	**128**	0.06
CRKP-Feces3	–	**≥128**	**≥64**	**≥64**	**≥64**	**≥64**	**≥16**	**≥16**	**≥4**	**≥8**	**≥512**	**>128**	**>128**	**>1024**	4/4	4	<0.03

^a^ +, positive; –, negative.

^b^ TZP, piperacillin/tazobactam; CTT, cefotetan; CAZ, ceftazidime; FEP, cefepime; AMK, amikacin; GEN, gentamicin; TOM, tobramycin; CIP, ciprofloxacin; LEV, levofloxacin; NIT, nitrofurantoin; IMP, imipenem; MEM, meropenem; FOS, fosfomycin; CAZ/AVI, ceftazidime/avibactam; TGC, tigecycline; CST, colistin. Numbers shown in bold were resistance judged by CLSI breakpoint.

### PFGE analysis and the location of carbapenem resistance genes

PFGE analysis revealed that nine of eleven isolates were grouped in the same PFGE type (clone A) except CRKP-Feces1 (clone B) and Feces2 (clone C) (Table S2). All strains were positive for the *bla*_KPC-2_ gene, which was located on a plasmid of approximately 100 kb in size (Figure S2).

### Phylogenetic and genotypic analysis of CRKP isolates

**Figure 2.**
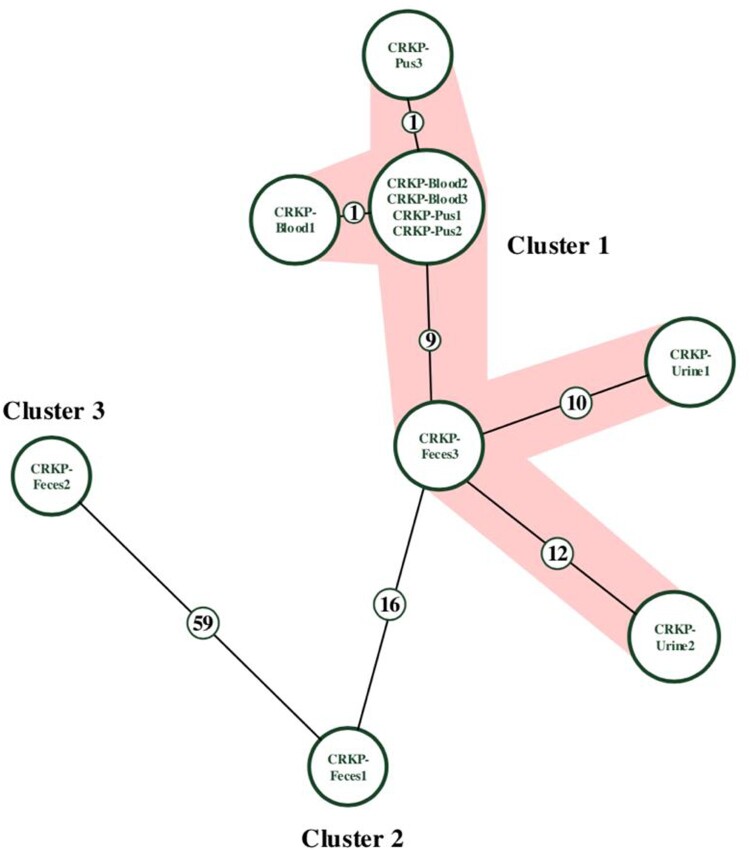
Minimum-spanning tree of cgMLST profiles among eleven CRKP isolates. Note: The minimum-spanning tree was generated based on cgMLST analysis with 2358 conserved genome-wide genes. A cluster was defined at a distance of ≤15 alleles.

Whole-genome sequencing revealed eleven CRKP isolates belonging to the same clone type, ST11. Nine were clustered into the same cgMLST group with fewer than 12 allelic differences, while CRKP-Feces1 and CRKP-Feces2 were clustered into groups 2 and 3, respectively (Figure 2 and Table S2).

Eight resistance genes, including *bla*_KPC-2_, *aadA2b*, *bla*_SHV-182_, *fosA*, *drfA14*, *sul2*, *qnrS1* and *tet*(A), were identified across the eleven strains, whereas five genes were variable (Table S2). Virulence factors related to adherence, antiphagocytosis, efflux pumps, iron acquisition, regulation, secretion systems and serum resistance were found, which revealed that they all belonged to the hypervirulent CRKP KL64 type (Table S2 and Figure S3).

### Genomic comparative analysis of ST11-KL64 hv-CRKP

Based on the high genome similarity and relatedness among these ST11-KL64 hv-CRKP strains, the first collected representative, CRKP-Urine1, of the main cluster harboured the most resistance and virulence genes. The isolate CRKP-Urine1 had three plasmids (pVi-CRKP-Urine1, pKPC-CRKP-Urine1 and pqnrS1-CRKP-Urine1). Virulence genes for RmpA and aerobactin were identified on pVI-CRKP-Urine1, with over 88% query coverage and over 99.95% identity to other virulence plasmids (e.g. MG053312, AP006726, and QURI01000002) (Figure S3B). The pKPC-CRKP-Urine1 plasmid belonged to the IncFII-IncR type and harboured the *bla*_KPC-2_ gene, which is almost identical to the plasmid pKPC-CR-hvKP-C789 (CP034417). Multiple resistance genes were carried by the plasmid pqnrS1-CRKP-Urine1, with a size of 85.2 kb, which possessed a backbone sequence similar to that of pLAP2_020079 (CP029382) (Figure S3B).

**Figure 3.**
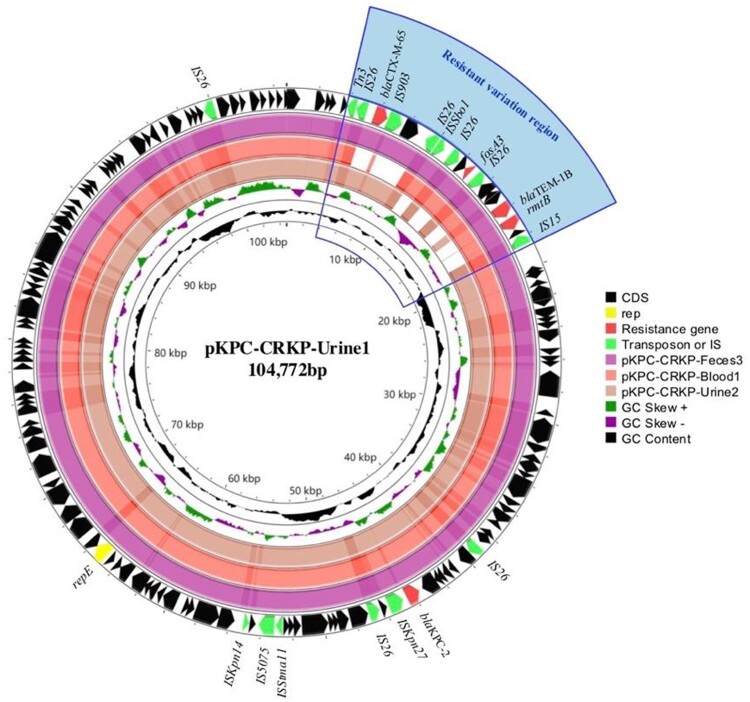
Sequence alignment of *bla*_KPC-2_ plasmids among CRKP-Urine1 and CRKP-Feces3, CRKP-Blood1 and CRKP-Urine2. Note: The resistance variation region was located at 4–19 kb of pKPC-CRKP-Urine1. Four copies of insertion sequence 26 (IS2*6*) and resistance genes were identified in the resistance variation region. The *bla*_TEM-1B_, *rmtB* and *fosA3* genes were absent in CRKP-Urine2, whereas *bla*_CTX-M-65_ was not found in CRKP-Blood1.

Notably, the *bla*_KPC-2_ plasmids among CRKP-Blood1, CRKP-Urine1, CRKP-Urine2 and CRKP-Feces3 showed high homology, with >94% coverage similarity, whereby variable sequences were identified at the region of resistance genes, and four copies of insertion sequence *26* (IS*26*) were 4–19 kb in length, indicating homologous recombination in this region (Figure 3).

### Point mutations are responsible for the resistance to tigecycline and colistin

When compared to CRKP-Urine1, several mutations and insertions were detected in the genomic sequences of the remaining tigecycline- or colistin-resistant isolates (Table 2). The gene alterations associated with tigecycline resistance were located only on *acrR*, *ramR*, *lon* and *tet(A)*. The *acrR* gene was interrupted by IS*26* in all the CRKP isolates. Amino acid mutations were identified in RamR at G152D and A37T, in Lon at V360G, G361R and K362stop, and in Tet(A) at G262D and A395V (Table 2). Among colistin resistance mutations, *pmrB*, *phoQ* and *mgrB* were identified with amino acid mutations of T157P, G385S and Q30stop, respectively (Table 2).

**Table 2.** Mutations of tigecycline and colistin resistance determinants in amino acid sequences.

Name	Tigecycline-resistant mutations^a^			Colistin-resistant mutations^b^			
	AcrR^c^	RamR	Lon	Tet(A)	PmrB	PhoQ	MgrB
CRKP-Urine1	+	–	–	–	–	–	–
CRKP-Blood1	+	–	–	+ (G262D)	–	–	–
CRKP-Blood2	+	–	–	+ (G262D)	–	–	–
CRKP-Blood3	+	–	–	+ (G262D)	–	–	–
CRKP-Urine2	+	+ (G152D)	–	–	–	+(G385S)	–
CRKP-Pus1	+	–	–	+ (G262D)	–	–	+ (Q30stop)
CRKP-Feces1	+	+ (G152D)	–	–	–	–	–
CRKP-Pus2	+	–	–	+ (G262D)	–	–	+ (Q30stop)
CRKP-Pus3	+	–	–	+ (G262D)	+ (T157P)	–	–
CRKP-Feces2	+	+ (G152D, A37T)	+ (V360G, G361R, K362stop)	+ (A395V)	–	–	–
CRKP-Feces3	+	+ (G152D)	–	–	–	–	–

^a, b^ G, Glycine; D, Aspartic Acid; A, Alanine; T, Threonine; V, Valine; R, Arginine; K, Lysine; P, Proline; S, Serine; Q, Glutamine.

^c^ The *acrR* gene was interrupted by the IS*Kpn26*.

### In-host evolution of resistance against tigecycline and colistin

**Figure 4.**
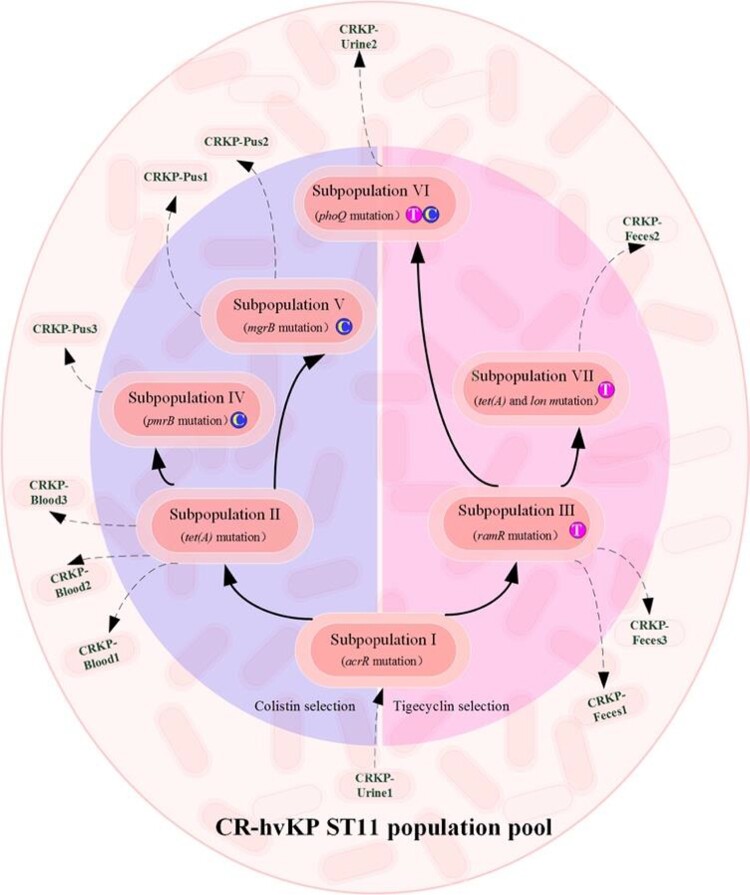
In-host resistance evolution pathways against colistin and tigecycline.

Resistance acquisitions of ST11-KL64 CRKP isolates due to antimicrobial agent selection were revealed by the mechanism of gene mutations, which indicated four potential evolution strategies, as shown in Figure 4. The strain CRKP-Urine1 was initially collected prior to treatment with both drugs, representing the nearest status to the preliminary CRKP population pool in the host. A secondary subpopulation II, evolved as a carrier of mutated TetA, contributed to the emergence of CRKP BSI. Soon after the elimination of the BSI pathogen by polymyxin B, colistin-resistant strains were isolated from cutaneous abscess samples and belonged to two subpopulations (IV and V), which suggests different evolution trends against colistin in this patient. In addition, evolution of resistance to tigecycline was observed in both the gastrointestinal and urinary tracts. Long-term selection of tigecycline promoted the subpopulation to evolve from III to VII for higher-level insusceptibility, with continuous CRKP isolates collected from feces. However, fecal carriage of CRKP could persist until one-month discharge period without antibiotic treatment and implied that the evolved subpopulation III ultimately colonized the gastrointestinal tract (Figure 4).

## Discussion

In China, ST11 is one of the most predominant clones of hospital-acquired carbapenem-resistant *K. pneumoniae* (HA-CRKP), which has aroused wide attention recently because an increasing number of clinical strains are hypervirulent and extremely drug resistant [33,34]. With the development of whole-genome sequencing, Zhou K. reported that CRKP of the ST11-KL64 type has gradually replaced ST11-KL47, being the most epidemic hypervirulent CRKP clone and causing the higher mortality of infected patients in China since 2016 [6]. On the condition that various infections mediated by CRKP isolates were reported previously [35,36], we further highlighted an ST11-KL64 CRKP-infected patient suffering from continuous and multisite infections.

To date, diagnostics and definition of hypervirulence of *K. pneumoniae* has still been a controversial issue with most potential determinants gradually been found in non-hypervirulent strains [37]. However, to begin with, the hypervirulent *K. pneumoniae* was originally recognized and defined through clinical characterizations of invasive infections. By this means, our ST11-KL64 CRKP not only caused multiple, periodic, distantly spread infections, but also colonized the gastrointestinal tract, even under the usage of tigecycline and polymyxin B, suggesting the potential abilities of hypervirulence. Further whole-genome sequencing provided more evidence of CRKP hypervirulence on both the chromosome and plasmids. Similar to other ST11-KL64 CRKP isolates, our isolates carried fourteen virulence factors on the chromosome and a typical virulence plasmid (pVi-CRKP-Urine1) with 95% similarity compared to the pK2044 plasmid [6] (Table S2, Figure S3). In particular, capsule genes play a role in inhibiting phagocytosis and immune evasion from host cells, as well as joining with type 3 fimbriae in biofilm formation to promote resistance to host killing and antimicrobials [38]. Meanwhile, hypervirulent *K. pneumoniae* (hvKP)-specific regulator (RmpA, RmpA2)-mediated overproduction of capsule, which contributes to the bactericidal activity of complement and antiphagocytosis, can help hvKP strains survive in the host and seize the chance to metastatically spread from the bloodstream [39]. In addition, plasmid-mediated aerobactin has been proven to account for up to 90% of the total four siderophores and notably improve hvKP fitness in human ascites and serum, of which genes were also found in our hv-CRKP strains [40]. Notwithstanding several cases considered that the carriage of virulence factors or a virulent plasmid was insufficient to identify the hvKP strain [41], most of our ST11-KL64 CRKP strains showed similar hypervirulence as the known hvKP NTUH-K2044 in the animal infection model. Furthermore, no single virulence factor has been identified to help determine hvKP with high sensitivity and specificity, both potential phenotypic and genotypic traits of hypervirulence should be concentrated for the ST11-KL64 hv-CRKP clone.

Under the trend of poor prognosis, sufficient antimicrobial agent therapies are urgently needed to monitor hv-CRKP infectious diseases. However, resistance to tigecycline and/or colistin could develop rapidly soon after clinical usage, which was observed in this report and further interpreted by mutations in common targets [17,42]. In contrast to previous studies [11,15,43], interruption of AcrR or Tet(A) mutants contributed less to increased tigecycline MICs. Apart from the most reported target RamR [44], of which mutants brought only moderate changes in MICs (4∼16 mg/L) in this study, a novel mutation in Lon protease might be meaningful regarding increased resistance, with an MIC of 128 mg/L. Furthermore, sporadically reported mutations of three genes (*pmrB*, *phoQ* and *mgrB*) even simultaneously occurred in our CRKP isolates with acquired colistin resistance, which suggested high chromosomal genetic adaptability among the same hv-CRKP clone. Moreover, genetic flexibility also existed in the plasmids. The *bla*_KPC-2_-carrying plasmid of our ST11-KL64 CRKP strains showed high genetic diversity in the MDR region, with resistance factors flanked by the IS*26* mobile element. In parallel, the high mobility of IS*26* could mediate the horizontal transmission of resistance genes or the functional deficiency of partial genes, which could be related to genetic adaptation of plasmids under antibiotic pressure [45,46].

During empirical therapies applied to infections, lasting antibiotic exposure could provide a selective advantage for acquired resistance in intestinal microecology [47]. In this study, the first fecal CRKP isolate was also obtained during the inpatient period. Even worse, the gastrointestinal colonization of CRKP has now become a neglected issue with a high risk of severe infection and mortality [48,49]. The last screening result indicated that fecal carriage of the same ST11-KL64 hv-CRKP clone could persist until the first month of discharge with elimination of infected strains, which might enable recurrence of infections. Similar cases have rarely been reported, but one case also exhibited prolonged CRKP rectal colonization in Zhang's study [50].

This study clarified that ST11-KL64-type hv-CRKP could cause severe infections with poor prognosis in a male patient, while its in-host resistance evolution on both chromosomes and plasmids could develop soon after the initiation of antimicrobial agents. Concerningly, similar genome sequences of hypervirulence and resistance plasmids among *K. pneumoniae* strains were widely submitted to the NCBI database, which indicates the potential menace of broad dissemination of ST11-KL64 hv-CRKP strains in the clinic.

## Supplementary Material

SUPPLEMENTAL_FILE_R1.docx
