# Supplementary material for: Resistance evolution of hypervirulent carbapenem-resistant Klebsiella pneumoniae ST11 during treatment with tigecycline and polymyxin
Source: Emerg Microbes Infect. 2021 Jun 13;10(1):1129–36. doi: 10.1080/22221751.2021.1937327 (PMC8205050; doi:10.1080/22221751.2021.1937327)
Supplement: SUPPLEMENTAL_FILE_R1.docx [file TEMI_A_1937327_SM8686.docx]

**SUPPLEMENTAL METERIAL**

**Table S1.** Confirmation primers for tigecycline or colistin resistance mutations

| Resistance phenotype | Gene | Name | Sequence (5’-3’) | Reference |
| --- | --- | --- | --- | --- |
| Tigecycline resistance | Efflux pump genes | *acrR*-F | AAACCCACTACAGTCTTTCTAT | This study |
|  |  | *acrR*-R | GCTAAAAATTCACGTTCATC |  |
|  |  | *ramR*-F | GATGGCGACCACGCTAAA |  |
|  |  | *ramR*-R | GCTCGGTAAACGGGTAGGT |  |
|  |  | *lon*-F | TCCCGCCGTTGAATGTGTGG |  |
|  |  | *lon*-R | ACTTACCAGCCCTATTTTTAT |  |
|  | *tet* gene | *tet(A)*-F | GCCTTTCCTTTGGGTTCTCT | ^1^ |
|  |  | *tet(A)*-R | TGTCCGACAAGTTGCATGAT |  |
| Colistin resistance | LPS modification genes | *pmrB*-F | TTAAGAAGGCCGTGCTATCC |  |
|  |  | *pmrB*-R | GATGAGGATAGCGCCCATGC |  |
|  |  | *phoQ*-F | ATACCCACAGGACGTCATCA | ^2^ |
|  |  | *phoQ*-R | CAGGTGTCTGACAGGGATTA |  |
|  |  | *mgrB*-F | AAGGCGTTCATTCTACCACC |  |
|  |  | *mgrB*-R | TTAAGAAGGCCGTGCTATCC |  |

**Table S2.** Genetic analysis of phylogenetic relationships, acquired antimicrobial resistance genes and virulence genes

| Name | Phylogenetic analysis | | | Different acquired antimicrobial resistance genes^b^ | Common virulence factor (related genes) ^c^ |
| --- | --- | --- | --- | --- | --- |
|  | PFGE | ST | cgMLST^a^ |  |  |
| CRKP-Urine1 | A | 11 | Cluster 1 | *bla*_CTX-M-65_, *fosA3*, *rmtB*, *bla*_TEM-1B_, *catA2* | Type 1 fimbriae (*fimA, fimB, fimC, fimD, fimE, fimF, fimG, fimH, fimI* and *fimK*), Type 3 fimbriae *(mrkA, mrkB, mrkC, mrkD, mrkF, mrkH, mrkI* and *mrkJ*), Capsule (*wzc* and *wzi*), AcrAB (*acrA and acrB*), **Aerobactin (*iucA, iucB, iucC, iucD* and *iutA*)**^d^, Ent siderophore (*entA, entB, entC, entD, entE, entF, entS, fepA, fepB, fepC, fepD, fepG* and *fes*), Salmochelin (*iroE* and *iroN*), Yersiniabactin (*fyuA, irp1, irp2, ybtA, ybtE, ybtP, ybtQ, ybtS, ybtT, ybtU* and *ybtX*), RcsAB (*rcsA, rcsB*), **RmpA (*rmpA2* and *rmpA*)**^d^, T6SS-I (*clpV/tssH, dotU/tssL, hcp/tssD, icmF/tssM, impA/tssA, ompA, sciN/tssJ, tli1, tssF, tssG, vasE/tssK, vgrG/tssI, vipA/tssB* and *vipB/tssC*), T6SS-II (*clpV*), T6SS-III (*icmF, impA, impF, impG, impH* and *sciN*), LPS rfb locus (*rfb*) |
| CRKP-Blood1 | A | 11 | Cluster 1 | *fosA3*, *rmtB*, *bla*_TEM-1B_, *catA2* |  |
| CRKP-Blood2 | A | 11 | Cluster 1 | *fosA3*, *rmtB*, *bla*_TEM-1B_, *catA2* |  |
| CRKP-Blood3 | A | 11 | Cluster 1 | *fosA3*, *rmtB*, *bla*_TEM-1B_, *catA2* |  |
| CRKP-Urine2 | A | 11 | Cluster 1 | *bla*_CTX-M-65_ |  |
| CRKP-Pus1 | A | 11 | Cluster 1 | *fosA3*, *rmtB*, *bla*_TEM-1B_, *catA2* |  |
| CRKP-Feces1 | B | 11 | Cluster 2 | *bla*_CTX-M-65_, *fosA3*, *rmtB*, *bla*_TEM-1B_ |  |
| CRKP-Pus2 | A | 11 | Cluster 1 | *fosA3*, *rmtB*, *bla*_TEM-1B_, *catA2* |  |
| CRKP-Pus3 | A | 11 | Cluster 1 | *fosA3*, *rmtB*, *bla*_TEM-1B_, *catA2* |  |
| CRKP-Feces2 | C | 11 | Cluster 3 | *bla*_CTX-M-65_, *fosA3*, *rmtB*, *bla*_TEM-1B_, *catA2* |  |
| CRKP-Feces3 | A | 11 | Cluster 1 | *bla*_CTX-M-65_, *fosA3*, *rmtB*, *bla*_TEM-1B_, *catA2* |  |

^a^ The CRKP strains based on core genome MLST (cgMLST) allelic profiles, 2358 conserved genome-wide genes were analysed, and minimum-spanning tree was generated by using SeqSphere+ software (Ridom GmbH, Muenster, Germany).

^b^ Resistance genes were identified by CGE tool (<https://cge.cbs.dtu.dk/services/ResFinder/>), which indicated that all of our eleven CRKP isolates were positive with *bla*_KPC-2_, *aadA2b*, *bla*_SHV-182_, *fosA*, *drfA14*, *sul2*, *qnrS1* and *tet*(A) genes.

^c^ Virulence genes were predicted by using online tools of VFanalyer (http://www.mgc.ac.cn/cgi-bin/VFs/v5/main.cgi?func=VFanalyzer) and Kaptive Web (<http://kaptive.holtlab.net/>).

^d^ The genes in bold were absent in the CRKP-Feces2 strain.

**Figure S1.** Virulence potential of CRKP strains in a *G. mellonella* infection model.

The effective of inoculum of 1×10^6^ CFU of bacteria was evaluated on the survival of *G. mellonella* larvae. Test group: CRKP-Urine1, CRKP-Urine2, CRKP-Feces1, CRKP-Feces3, CRKP-Blood1, CRKP-Blood3, CRKP-Pus1, CRKP-Pus3; Positive control group: hvKP NTUH-K2044; Negative control group: cKP04017; Blank control group: Injection with equal volume of PBS.


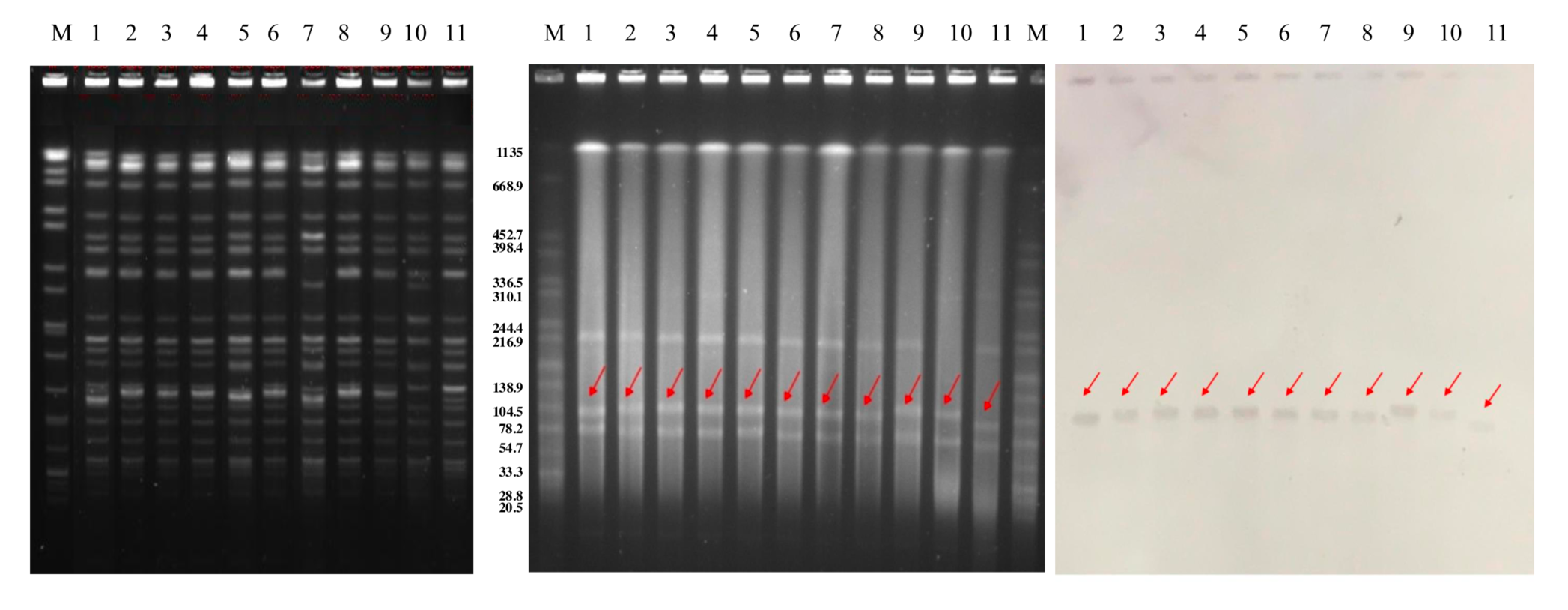


**Figure S2.** PFGE, S1-PFGE and Southern blot with *bla*_KPC-2_ probe.

M, *Salmonella choleraesuis* H9812 (MIS00418); 1, CRKP-Urine1; 2, CRKP-Urine2; 3, CRKP-Blood1; 4, CRKP-Blood2; 5, CRKP-Blood3; 6, CRKP-Pus1; 7, CRKP-Pus2; 8, CRKP-Pus3; 9, CRKP-Feces3; 10, CRKP-Feces2; 11, CRKP-Feces1


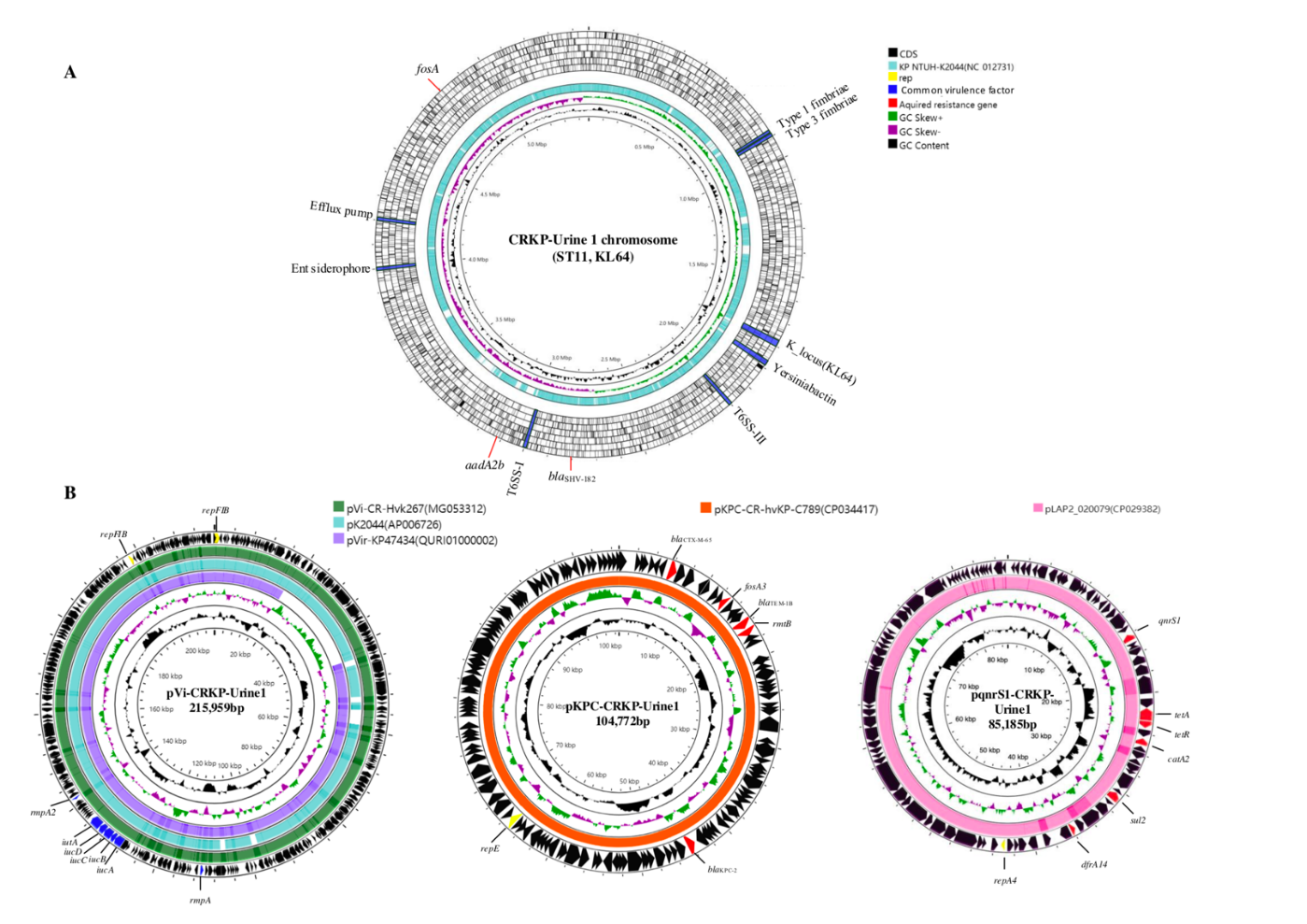


**Figure S3.** Genomic comparison and characteristics of CRKP-Urine1 for virulence and resistance genes.

1. The complete chromosomal genome of CRKP-Urine1 was compared to the chromosomal genome of NTUH-K2044 (NC 012731). High diversity of virulence factors (type 1 fimbriae, type 3 fimbriae, yersiniabactin, T6SS-I, T6SS-III, Ent siderophore and efflux pump) and three resistance genes (*bla*_SHV-182_, *aadA2b*, and *fosA*) are shown.
2. The strain CRKP-Urine1 has one virulence plasmid and two resistance plasmids. The virulence plasmid (pVi-CRKP-Urine1) has seven virulence genes (i.e., *rmpA*/*rmpA2*, *iucA/B/C/D*, and *iutA*), with a size of 216.0 kb, which presents high homology with three virulence plasmids, pVi-CR-Hvk267 (MG053312), pK2044 (AP006726) and pVir-KP47434 (QURI01000002). The two resistance plasmids (pKPC-CRKP-Urine1 and pqnrS1-CRKP-Urine1) have eleven resistance genes, *bla*_KPC-2_, *bla*_CTX-M-65_, *bla*_TEM-1B_, *rmtB*, *fosA3*, *qnrS1*, *sul2*, *drfA14*, *catA2*, *tet*A, and *tet*R, which possess backbone sequences similar to those of the resistance plasmids pKPC-CR-hvKP-C789 (CP034417) and pLAP2_020079 (CP029382), with both 100% coverage and 100.00% identity, respectively.

**References**

1 Chiu S, Huang L, Chen H *et al.* Roles of ramR and tet(A) Mutations in Conferring Tigecycline Resistance in Carbapenem-Resistant Klebsiella pneumoniae Clinical Isolates. *Antimicrob Agents Chemother* 2017; **61**: 1–10.

2 Quan J, Li X, Chen Y *et al.* Prevalence of mcr-1 in Escherichia coli and Klebsiella pneumoniae recovered from bloodstream infections in China: a multicentre longitudinal study. *Lancet Infect Dis* 2017; **17**: 400–410.
